# Prospects for exploiting epigenetic effects in livestock production

**DOI:** 10.1093/af/vfab071

**Published:** 2021-12-17

**Authors:** Flavio S Schenkel

**Affiliations:** Centre for Genetic Improvement of Livestock, Department of Animal Biosciences, University of Guelph, Guelph, ON N1G 2W1, Canada

In the last couple of years, the interest in possible applications of epigenetics aiming to improve livestock production and welfare has grown. The Canadian Society of Animal Science is proud to present this issue of *Animal Frontiers* focused on the prospects for exploiting epigenetic effects in livestock production. Ten articles highlighting how epigenetics may impact livestock production in the future are included in this issue of *Animal Frontiers*. The contributions from Asia, Europe, North America, Oceania, and South America provide a global perspective of how livestock scientists view the prospects for exploiting epigenetic effects in the future.

Livestock animals are expose to many environment-related factors during a production cycle, which interact with the epigenome and the underlying genome to determine phenotypic expression. Ibeagha-Awemu and Yu (2021) show that epigenetic factors are involved in the modulation of livestock reproduction, growth, productivity, and health, and are additional layers of information that can be exploited for livestock management and improvement. Exploring these non-Mendelian and non-DNA-based inheritance mechanisms, especially the DNA methylation inheritance, could help to understand better how phenotypes are determined and used for management and selection decisions, as reviewed by Zhang and Sirard (2021). Genomic selection and the use of elite sires are important tools for improving traits in the context of livestock breeding. However, performances are also impacted by environmental factors, thus limiting the efficiency of genomic selection. Kiefer et al. (2021) argue that, because epigenetic mechanisms translate the environmental effects at the genome level, they are a potential source of phenotypic variance currently unaccounted by genomic selection. The authors show that several studies conducted in humans or model species have established the role of the in utero conditions in the long-term programming of phenotypes. More recently, the importance of the paternal epigenome in the determination of offspring phenotypes has been demonstrated. Mi et al. (2021) point out that the identification of sperm-specific DNA methylation markers at different time points is important for improving the phenotypic performance of domestic animals, including cattle and pigs. DNA methylation can regulate sire fertility and semen quality and influence offspring phenotypes. The effects of inter/trans-generational inherited DNA methylation information induced by paternal experience and environmental stimuli are significant in farm animals. Furthermore, Ouellet et al. (2021) reveal that maternal response to heat stress alters developmental programming in lactating dairy cattle. Exposure of a late-gestation cow to environmental heat stress affects her offspring up to at least 4 yr after the heat insult. The authors show recent evidence indicating that differential DNA methylation arising in utero and intrauterine growth restriction are in part responsible for the long-term phenotypic changes. The establishment and proper regulation of the embryo development depend on several conditions that include changes in the chromatin structure, epigenetic modifications, protein modifications, activation of transcription factors and retrotransposon, and genome repair and stability. de Macedo et al. (2021) indicate that failures in the proper regulation of these events can result in either embryo development arrest and death, or embryo survival, but resulting in embryos having lower potential to continue developing, implanting, and producing a normal offspring. Costa et al. (2021) show that the intrauterine environment is crucial for the skeletal muscle formation, which depends on maternal supplies for an adequate growth and development. Disturbances involving, for instance, maternal feed restriction or overfeeding, directly affect the offspring’s skeletal muscle composition, influencing the final meat quality. The nutritional manipulation during the intrauterine period contributes to achieve desirable meat quality traits, such as marbling and tenderness. Metabolism plays an important role, providing metabolites that are used as substrates for epigenetic mechanisms (see Infographic). The microbiota could also play an important role in determining the epigenome landscape of an animal. The microbiota is a complex environment that leads to deep physiological and molecular changes in the host organism. Dunislawska et al. (2021) showed that the in ovo stimulation is a powerful, but still underestimated, method to control the microbiota in poultry and introduce epigenetic modifications at different levels. Alternate bioactive compounds stimulate alternate sets of genes in different genotypes. Consequently, it allows virtually unlimited modulation of many desired traits in poultry, driven by the intestinal microbiota reprogramming. The knowledge of how an animal’s environment can affect epigenetic marks of interest has the potential to allow producers to create optimal environments for their desired phenotypes. Pacht et al. (2021) described how environmental factors, such as dietary nutrients and stress from heat and transportation, impact DNA methylation. Altered DNA methylation was shown to impact livestock phenotypes including egg-laying, fiber growth, heat stress, mastitis, meat and milk production. Clarke et al. (2021) reason that the advancement in sequencing technologies is enhancing functionally annotated genomes in parallel to genomic selection strategies and that DNA methylation can be exploited as a molecular tool in livestock species through the use of epigenetic clocks to improve health, longevity, productivity, and environmental adaptation. However, to incorporate DNA methylation data into routine genetic evaluations, high throughput, robust and cost-effective assays still need to be developed.

Therefore, this issue of *Animal Frontiers* shows that there are growing opportunities for research into the direct and indirect links between environment, epigenome, genome and the ultimate phenotypes, and for application of this knowledge to improved livestock production and welfare. In due course, the livestock industry will have the increased ability to tailor the animal’s epigenome to produce phenotypes that are more desirable, while increasing animal welfare and resiliency.

## About the Author

Flavio S. Schenkel is a Professor at the University of Guelph, where he is the Director of the Centre for Genetic Improvement of Livestock and was the 2020/2021 President of the Canadian Society of Animal Science. He serves on a number of influential academic and industry boards in Canada. His research interests range from theoretical to applied genetics and genomics in livestock breeding, with a current focus on the use of genomic information to enhance the genetic evaluation of livestock species with emphasis on genomic selection.
